# Risk factors related with avascular necrosis after internal fixation of femoral neck fractures in children: a systematic review and meta-analysis

**DOI:** 10.3389/fped.2023.1188179

**Published:** 2023-08-02

**Authors:** Bo-Hai Qi, Xiao-Wei Wang, Xiao-Ming Wang, Huan Wang, Ya-ting Yang, Qiang Jie

**Affiliations:** ^1^ Pediatric Orthopedic Hospital, Honghui Hospital, Xi’an Jiaotong University, Xi’an, China; ^2^ Xi'an Medical University, Xi'an, China

**Keywords:** femoral neck fracture, AVN, children, risk factors, internal fixation, meta-analysis

## Abstract

**Background:**

Less than 1% of children develop femoral neck fractures (FNF), making them uncommon. However, they may have dangerous side effects, like avascular necrosis. Even though several risk factors for postoperative avascular necrosis have been identified, there is still debate regarding them. In this investigation, a meta-analysis was performed to examine the potential causes of postoperative avascular necrosis in children with FNF.

**Methods:**

We conducted a thorough literature search to find risk factors for avascular necrosis (AVN) after internal fixation of pediatric FNF. Until December 2022, we searched several databases, including PubMed, Embase, Cochrane Library, Web of Science, CNKI, Orthosearch, and Sinomed. Software Zotero 6.0 and Stata 17.0 were used to organise and synthesise the data. Finally, a sensitivity and publication bias test was carried out.

**Results:**

Our study includes a total of 15 case-control studies involving 814 patients. The risk of postoperative AVN increased with age at fracture encounter (95% CI: 0.64–1.88, *P* = 0.0003), initial fracture displacement (95% CI: 1.87–9.54, *P* = 0.0005), and poor fracture reduction (95% CI:1.95–22.34, *P* = 0.0024) were risk factors for postoperative AVN. There was no significant relationship between gender and postoperative AVN (95% CI: 0.52–1.31, *P* = 0.41). Conversely, Postoperative AVN and reduction methods have no connection with each other (95% CI: 0.77–2.66, *P* = 0.25), procedure time (95% CI: 0.43–2.99, *P* = 0.16), or injury mechanism (95% CI: 0.32–2.26, *P* = 0.75). The incidence of post-operative AVN varies between Delbet fracture types (95% CI: 0.15–0.31, *P* < 0.0001), with the overall trend being that the incidence of post-operative AVN is highest for type II, lowest for type IV, and close for types I and III, but it is not clear which type of fracture is the independent risk factor. Funnel plots indicate no significant publication bias.

**Conclusions:**

In line with this study, About 26% of children who underwent surgery for a femoral neck fracture suffered postoperative AVN. The main risk factors for AVN were the child's age, the initial displacement of the fractures, and poorly reduced fractures. The risk of AVN did not significantly correlate with gender, the time of the procedure, reduction methods or the mechanism of injury. The overall trend in the incidence of postoperative AVN for the different Delbet types of fracture is that the incidence of postoperative AVN is highest for type II, lowest for type IV, and close for types I and III, but it is not clear which type of fracture is the independent risk factor.

## Introduction

Less than 1% of cases of high-energy accidents to schoolchildren result in fractures of the neck of the femur, which are rare but serious injuries (1–4). AVN is a frequent side complication postoperatively to heal these fractures. The prognosis is poor and can rapidly result in the downturn of the femoral head if treatment is ineffective. This can damage the child's physical and emotional health and strain the family and society. It can also result in osteoarthritis of the hip joint during adolescence and may ultimately necessitate a hip replacement. For these fractures, there aren't any proven predictors or treatments.

Recently, researchers have attempted to identify the impacts of AVN in children with postoperative FNF. However, the outcomes of these investigations might be more reliable and conclusive. This study aims to collect data from published literature on AVN obeying pediatric FNF, analyse the data using meta-analysis, and assess the importance of the probable risk factors. This information will help prevent and treat this complication.

## Methodology and information

### Search procedure

The PRISMA guidelines in the conduct of this investigation (5) and the Epidemiological Observational Studies Meta-Analysis Group (6). The study was based on previously available data, so neither patient permission nor ethics approval was required. Two researchers (BHQ and XWW) searched for pertinent literature on postoperative AVN in children with FNF. From its commencement through December 2022, the examination encompassed various data, including PubMed, Embase, Cochrane Library, Web of Science, CNKI, Orthosearch, and Sinomed.

Medical subject headings (MeSH) and non-MeSH terms were used in both English and Chinese for the search, including keywords such as “children”, “pediatric”, “adolescents”, “hip fracture”, “femoral neck fracture”, “internal fixation”, “osteonecrosis of the femoral head” or “ONFH” or “avascular necrosis” or “AVN”. If there is any uncertainty or disagreement, discuss it and find a solution together, possibly with a third investigator. For instance, Table 1 displays the literature search approach for the PubMed database.

**Table 1:** Literature search keywords and search formulae.

#1 “Femoral Neck Fractures” [Mesh]; #2 Femur Neck Fracture; #3 Femur Neck Fractures; #4 Femoral Neck Fracture; #5 Femoral Neck Fractures
#6 #1 OR #2 OR #3 OR #4 OR #5
#7 “Fracture Fixation” [Mesh]; #8 Internal fixation; #9 sliding hip screws; #10 cancellous screws
#11 #7 OR #8 OR #9 OR #10
#12 “Femur Head Necrosis” [Mesh]; #13 Femur Head Necrosis; #14 osteonecrosis of the femoral head; #15 Avascular Necrosis of Femur Head: #16 ONFH; #17 AVN
#18 #12 OR #13 OR #14 OR #15 OR #16 OR #17
#19 “Child” [Mesh]); # 20 children
#21 #19 OR #20
#22 “Adolescent” [Mesh]); #23 Male Adolescents; #24 Male Adolescent; #25 Adolescent, Male; #26 Adolescents, Male; #27 Female Adolescents; #28 Female Adolescent; #29 Adolescent, Female; #30 Adolescents, Female; #31 Youths; #32 Youth; #33 Teenager; #34 Teenagers; #35 Teen; #36 Teens; #37 Adolescence; #38 Adolescents
#39 #22 OR #23 OR #24 OR #25 OR #26 OR #27 OR #28 OR #29 OR #30 OR #31 OR #32 OR #33 OR #34 OR #35 OR #36 OR #37 OR #38
#6 AND #11 AND #18 AND #21 AND #39

### Case selection

To gather pertinent information on the risk factors of AVN after children's FNF, two researchers (BHQ and XWW) independently analysed articles. The studies' inclusion criteria were: (1) Study type: Original studies may be case-control studies, cohort studies, cross-sectional research, randomised controlled trials, or non-randomized controlled trials. (2) Study population: Children and adolescents under 18 with subsequent traumatic, non-pathological FNF who underwent internal fixation procedures within two weeks of the injury constitute the study population. (3) Intervention: Internally fixed surgery with at least a six-month follow-up. (4) Outcome indicators: AVN after an FNF in children, covering the number of cases with various age ranges, genders, fracture types, initial displacements, reduction quality, reduction method, injury mechanism, and procedure timing.

The exclusion criteria were: (1) studies on patients who underwent internal fixation of FNF before 2000. (2) Studies with a patient population included patients with bilateral inter-rotor fractures, sub-rotor fractures, femoral stem fractures, tumour metastases, or long-term high-dose hormone use. (3) Study types include case reports, animal studies, reviews, conference papers, and studies with full texts or sufficient data. (4) Herbal therapy and autologous vascular and bone flap transplantation are examples of treatment options. (5) Studies utilising a sample size of 20 cases or less. (6) Duplicate publication of study data. (7) Studies with poor or moderate literature quality ratings. The researchers and corresponding author would discuss differences and reach a consensus.

### Data extraction

Two researchers (BHQ and XWW) used a standardised form to collect and organise data from the selected studies. The discussion was used to settle any disagreements. A standardised table was used to record the study's design, demographic characteristics (such as average age and length of follow-up), and relevant risk variables. To ensure accuracy, the data was carefully reviewed and verified. The results of the study are presented in a PRISMA flow chart.

### Methodological quality assessment

Two researchers (BHQ and XWW) evaluated the papers' quality in this study using the modified Newcastle–Ottawa Scale (NOS) (7). In the final standings, a third researcher (Qiang Jie, the corresponding author) made the ultimate determination. They each assigned a score based on a set of criteria. Studies scoring 6–9 out of a potential nine stars were considered relatively high quality. The studies included in this study were supposed to be of high quality, as they each received a score of 6 stars or higher.

### Statistical analysis

For the statistical analysis and meta-analysis, Stata 17.0 was employed. The odds ratio (OR) was utilised for statistical analysis, and the impact of heterogeneity on the outcomes of the meta-analysis was evaluated using the I-square (*I*^2^) test. The fixed-effect model was preferred if the *P*-value was more significant than 0.1 and the *I*^2^ was lower than 50%, demonstrating no statistical heterogeneity among the studies. But in cases where there was much heterogeneity, the random-effects model was utilised. To evaluate the consistency of the findings, a sensitivity analysis was performed by excluding each study separately. Statistical significance was defined as a *P*-value of 0.05. Data conversion, the funnel plot, and Egger's and Begg's tests were used to assess the publication bias in research that omitted important data (8).

## Results

### Study selection process

The selection process began with 940 references. After removing duplicates (407 contacts) and those not meeting the inclusion criteria (107 references), 351 authorities remained. After carefully examining the remaining references, 60 articles were eliminated because they contained dubious data, were published more than once, or were judged to be of moderate or low quality. Finally, the study included 15 articles that satisfied all the criteria. Table 2 provides a summary of the selection procedure's specifics.

**Table 2:** Preferred reporting items flowchart for systematic reviews and meta-analyses.

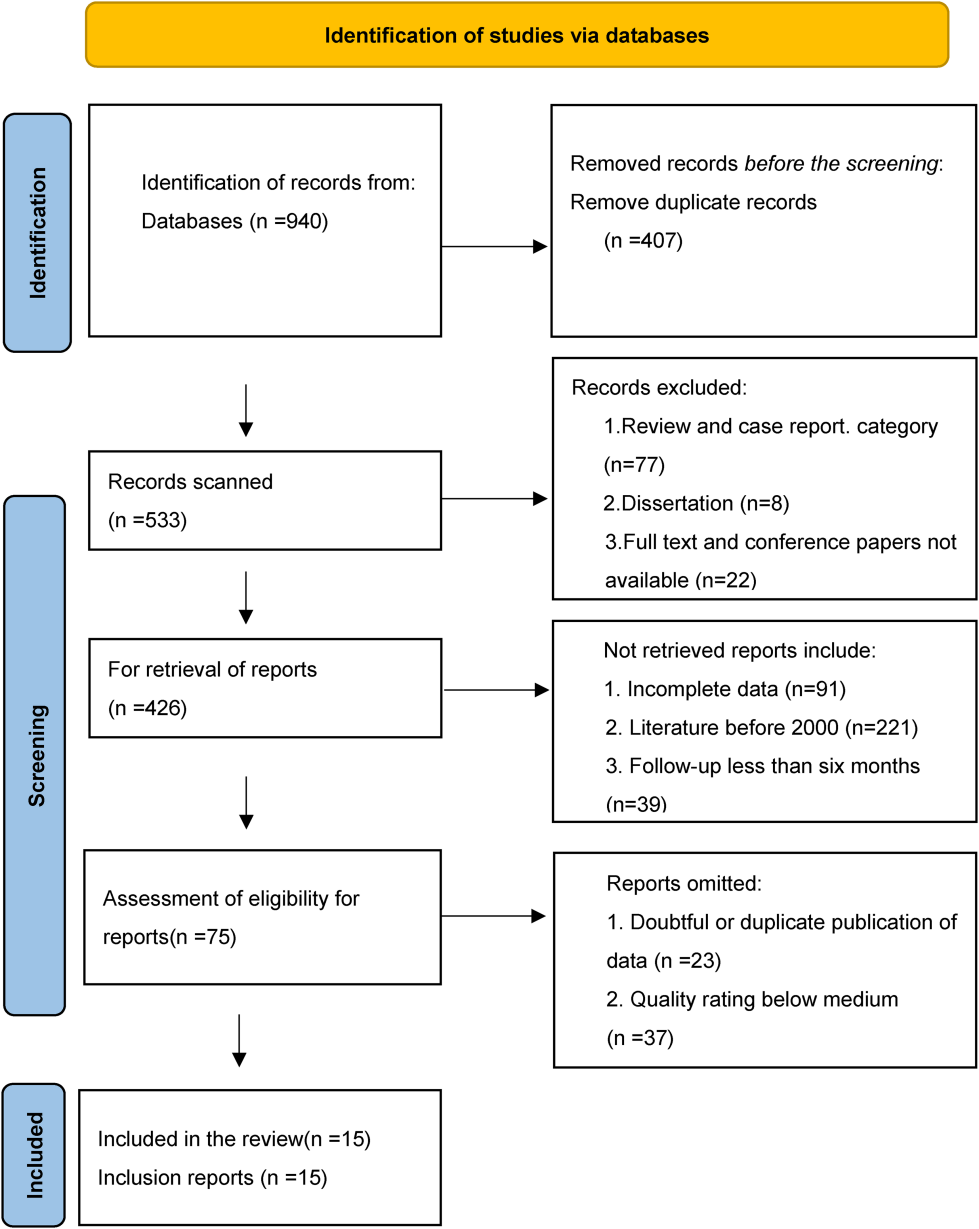

### Basic characteristics and assessment of the listed studies' quality

It should be noted that all 15 case-control studies included in the research selection process were published between the years 2008 and 2022. The research was conducted in several nations, including China (7 studies), the United States (3 studies), India (2 studies), Turkey (2 studies), and Serbia (1 study). The sample size of the selected clinical trials varied, ranging from 21 to 241 patients. 51.96 per cent of the patients with FNF were men. All included studies had a minimum follow-up period of six months, with the most extended period being 314.4 months. 215 patients in total suffered AVN. The three primary internal fixation techniques were proximal femoral splint internal fixation with open and closed reduction, hollow screw internal fixation, and hollow screw and smooth K-wires internal fixation. Utilising the modifications, the studies' quality was assessed.

All the listed studies received six stars or better on the Newcastle–Ottawa Scale. Table 3 displays the studies' broad information.

**Table 3:** Basic characteristics of the included studies.

Author	Year	characteristics	No. of cases	Follow-up visits (mo.)	Risk Factor	NOS score
Wu (9)	2019	10 males, 1–14 years	21	10–58	1, 3, 4, 8	7★
Wang (10)	2019	136 males, 2–17 years	241	6–86	1, 3, 4, 6, 8	8★
Wang (11)	2022	86 males, 3–17 years	153	6–86	1, 2, 3, 4, 6, 7, 8	8★
Varshney (12)	2009	14 males, 5–15 years	21	66–129	6, 8	6★
stone (13)	2015	13 males, 4.5–17.4 years	22	12–104.4	1, 2, 3, 5, 6, 7	8★
Spence (14)	2016	41 males, 1.3–18.1 years	72	Average 33.5	1, 2, 3, 4, 8	8★
Bukva (15)	2015	16 males, 4–14 years	28	48–216	8	8★
Baysal (16)	2016	16 males, 2–16 years	28	15–217	1, 2, 3, 4, 6, 8	8★
Dai (17)	2020	27 males, 2–14 years	44	11–224	1, 2, 3, 4, 5, 6, 7	8★
Riley (18)	2015	28 males, 0.9–17 years	44	12–314.4	1, 3, 5, 6, 8	9★
Xu (19)	2008	18 males, 3.7–14.1 years	33	12–146.4	1, 3, 4, 5	8★
Chaudhary (20)	2021	13 males, 5–16 years	21	12–20	1, 2, 3, 6, 8	7★
Li (21)	2022	21 males, 4.4–14 years	28	12–36	2, 3, 4, 5, 7, 8	9★
Chen (22)	2017	16 males, 4–16 years	24	6–48	3, 5, 6, 8	7★
Akar (23)	2022	25 males, 1–17 years	34	24–98	4, 7	7★

1 age; 2 genders; 3 Delbet fracture type; 4 initial fracture displacement; 5 quality of reduction; 6 methods of reduction; 7 mechanisms of injury; 8 the timing of the procedure.

## Meta-analysis results

### Risk factors associated with postoperative AVN

#### Gender

**Figure 1:**
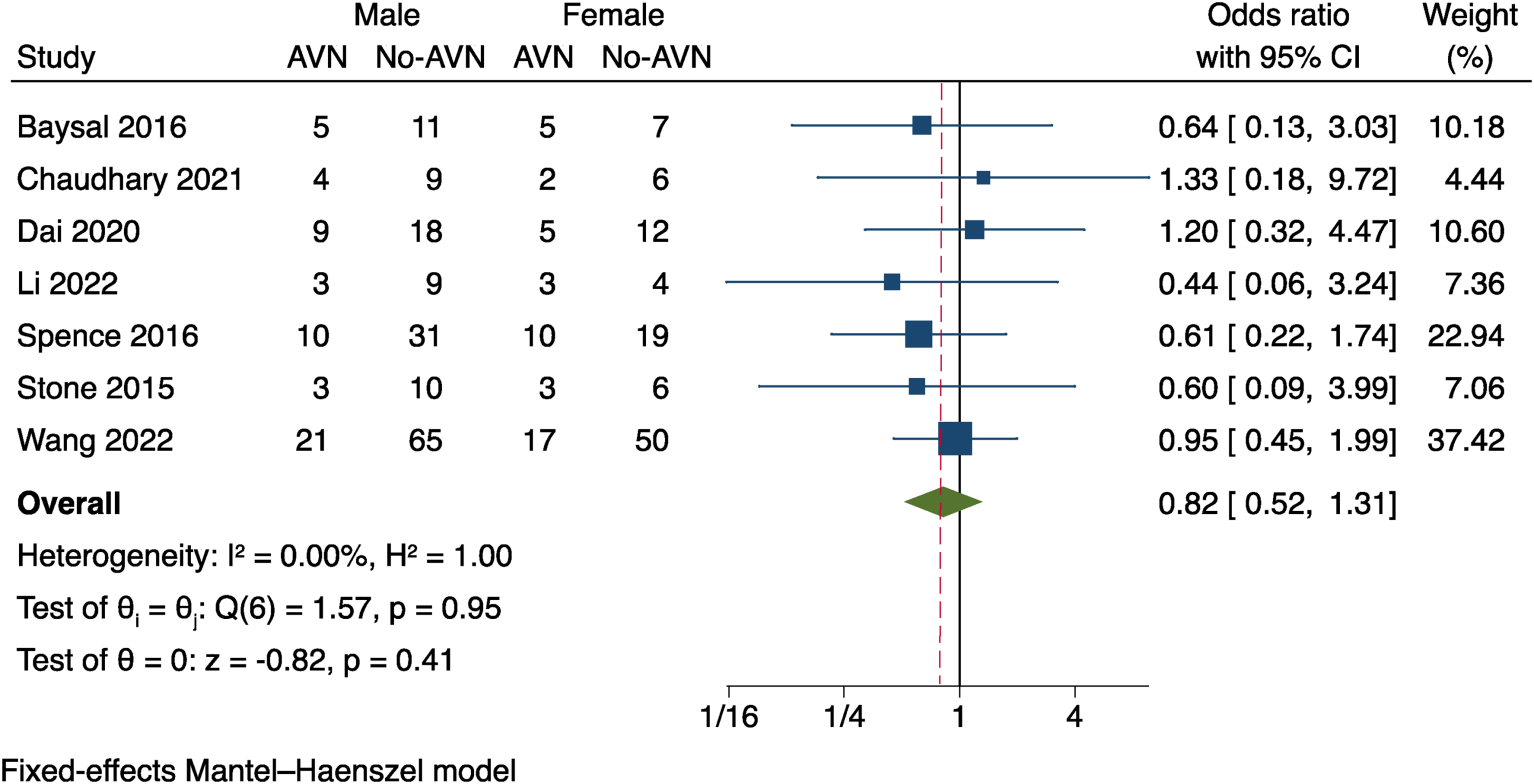
Results of the meta-analysis of the relationship between gender and postoperative AVN.

Seven of the 15 studies (9–15) included in the analysis evaluated the relationship between gender and AVN. Since these studies had no significant heterogeneity, a fixed effects model was utilised to analyse data (*I*^2^ = 0%, *P* = 0.95). Figure 1 illustrates the results, which revealed no statistically significant gender difference between patients with AVN and those without AVN (OR = 0.82, *P* = 0.41, 95% CI: 0.52–1.31).

#### Age

**Figure 2:**
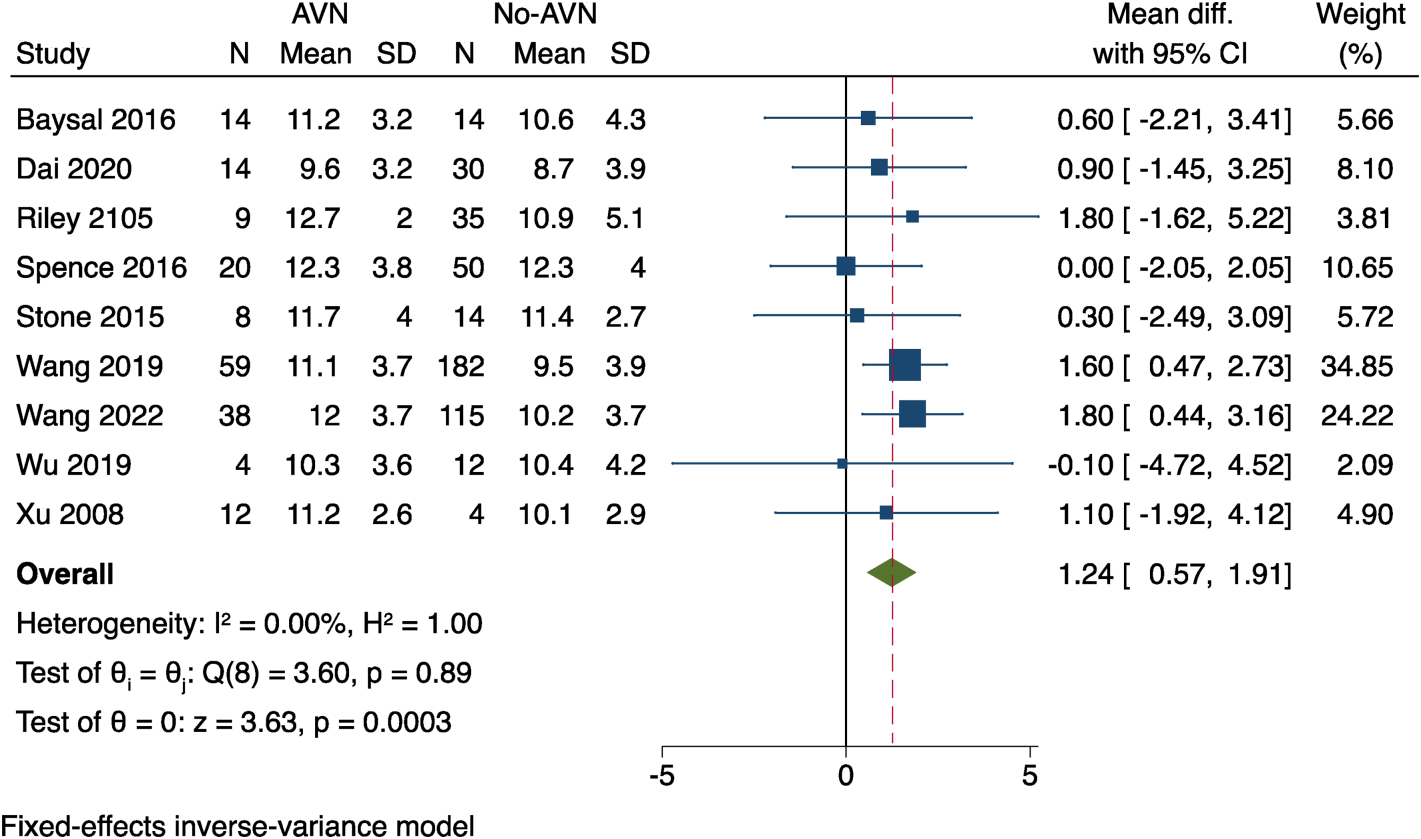
Results of a meta-analysis on the relationship between age and postoperative AVN.

There were nine studies (9–11, 13, 14, 16–19); Patients' ages, as well as a mean and standard deviation, were recorded in the raw data by the study data. Utilising a weighted mean difference (WMD), a meta-analysis was conducted, and it presented no proof of study heterogeneity (*I*^2^ = 0%, *P* = 0.89). The results showed that the 95% confidence interval was 0.64–1.88, with a *P*-value of 0.0003. According to these results, individuals who experienced AVN after surgery were older than those who did not, as shown in Figure 2.

### Delbet typing of fractures

**Figure 3:**
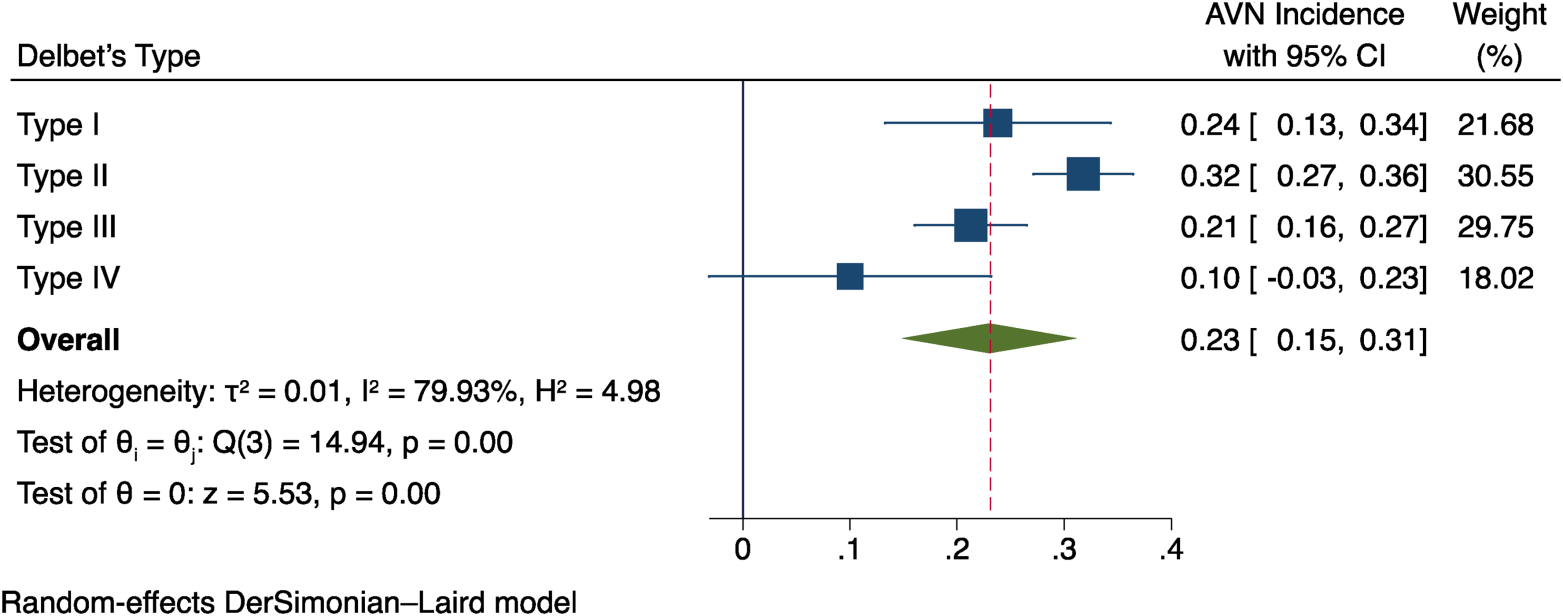
Findings from a meta-analysis of the relationship between postoperative AVN and fracture delbet type.

The data on fracture Delbet typing was available in 12 studies (9–11, 13, 14, 16–22) and was analysed through Meta-analysis. Two studies (13, 22) did not report any cases of type I fractures, and six studies (9–11, 17, 19, 20) did not report any cases of type IV fractures. Type II and III fractures were reported in all included studies. Due to heterogeneity among studies, a random-effects model was used (*I*^2^ = 79.93%), as shown in Figure 3. The findings showed a 23 per cent overall incidence of postoperative AVN (95% CI: 0.15–0.31, *P* < 0.0001). The results indicate that AVN occurs after different types of Delbet fractures, and the incidence rate varies statistically. The overall trend is that the incidence of AVN after type II fractures is the highest, and type IV fractures are the lowest, Types I and III are close, but it cannot be determined which type of fracture is an independent factor for postoperative AVN.

### Initial fracture displacement

**Figure 4:**
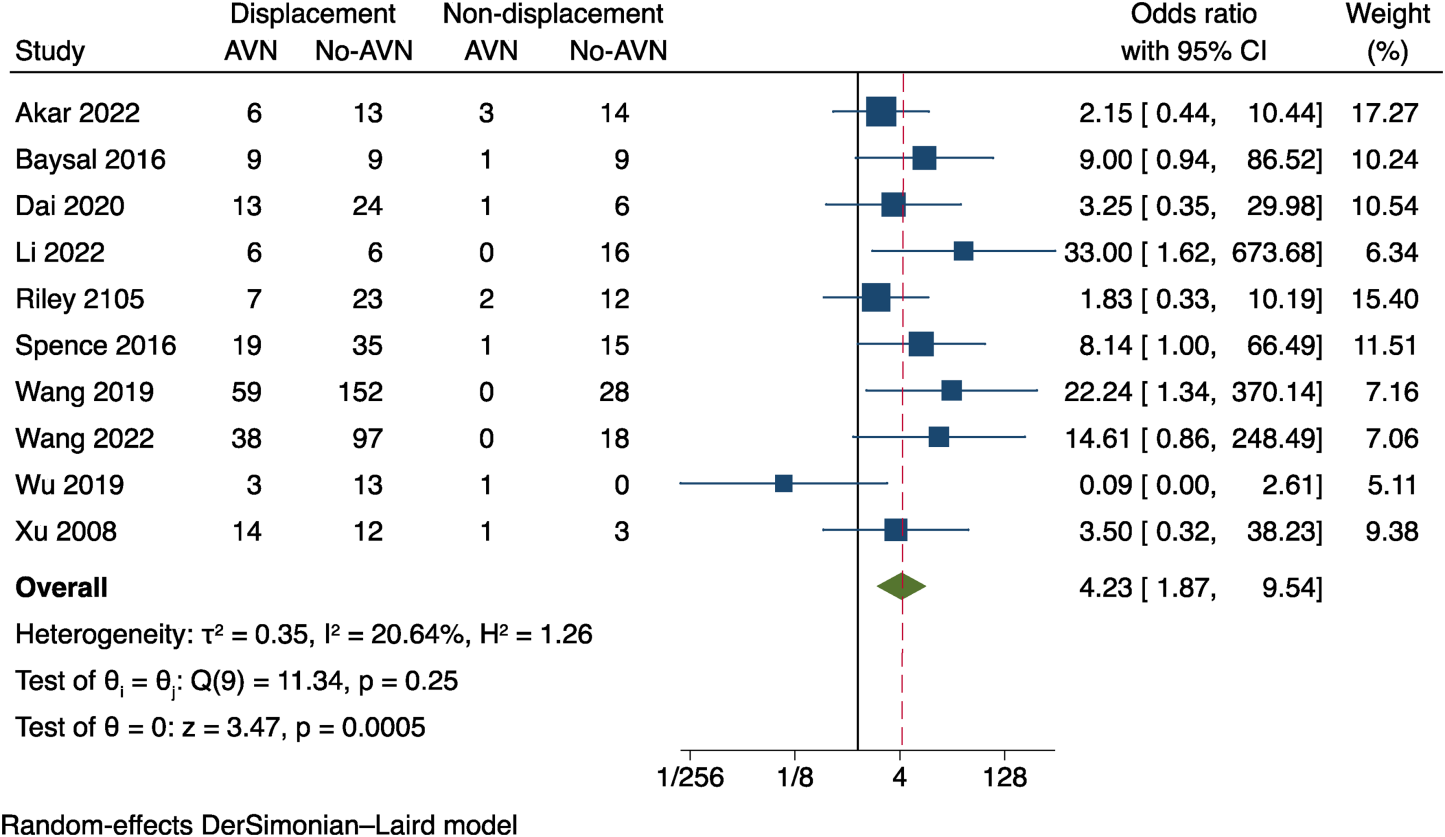
Results of a meta-analysis on the connection between initial fracture displacement and postoperative AVN.

The data on initial fracture displacement from 10 studies (9–11, 14, 16–19, 21, 23) was analysed. The results indicate a mild heterogeneity between the studies (*I*^2^ = 20.64%, *P* = 0.25) was applied, as seen in Figure 4. The information shows that a risk factor for postoperative AVN is the initial fracture displacement (OR = 4.23, 95% CI: 1.87–9.54, *P* = 0.0005).

### Quality of reduction

**Figure 5:**
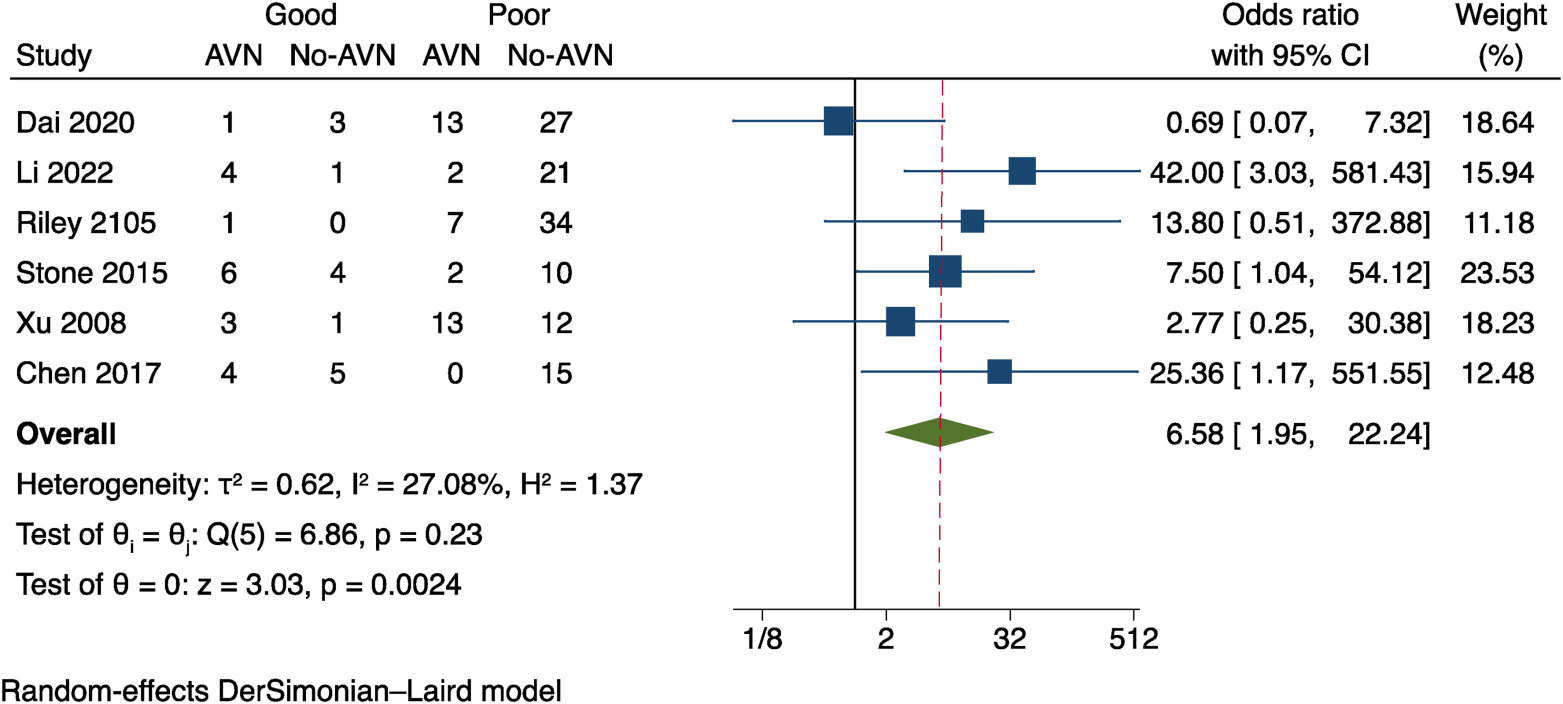
Results of a meta-analysis on the relationship between postoperative AVN and reduction quality.

Six studies (13, 17–19, 21, 22) were the subject of our meta-analysis. To evaluate the quality of fracture reduction. *I*^2 ^= 27.08%, *P* = 0.23 was used, and the results demonstrate a mild heterogeneity amongst the studies, as shown in Figure 5. The results revealed a statistical difference between the two groups (OR = 6.58 95% CI: 1.95–22.34, *P* = 0.0024). These results suggest that poor fracture reduction may raise the risk of postoperative AVN.

### Reduction methods

**Figure 6:**
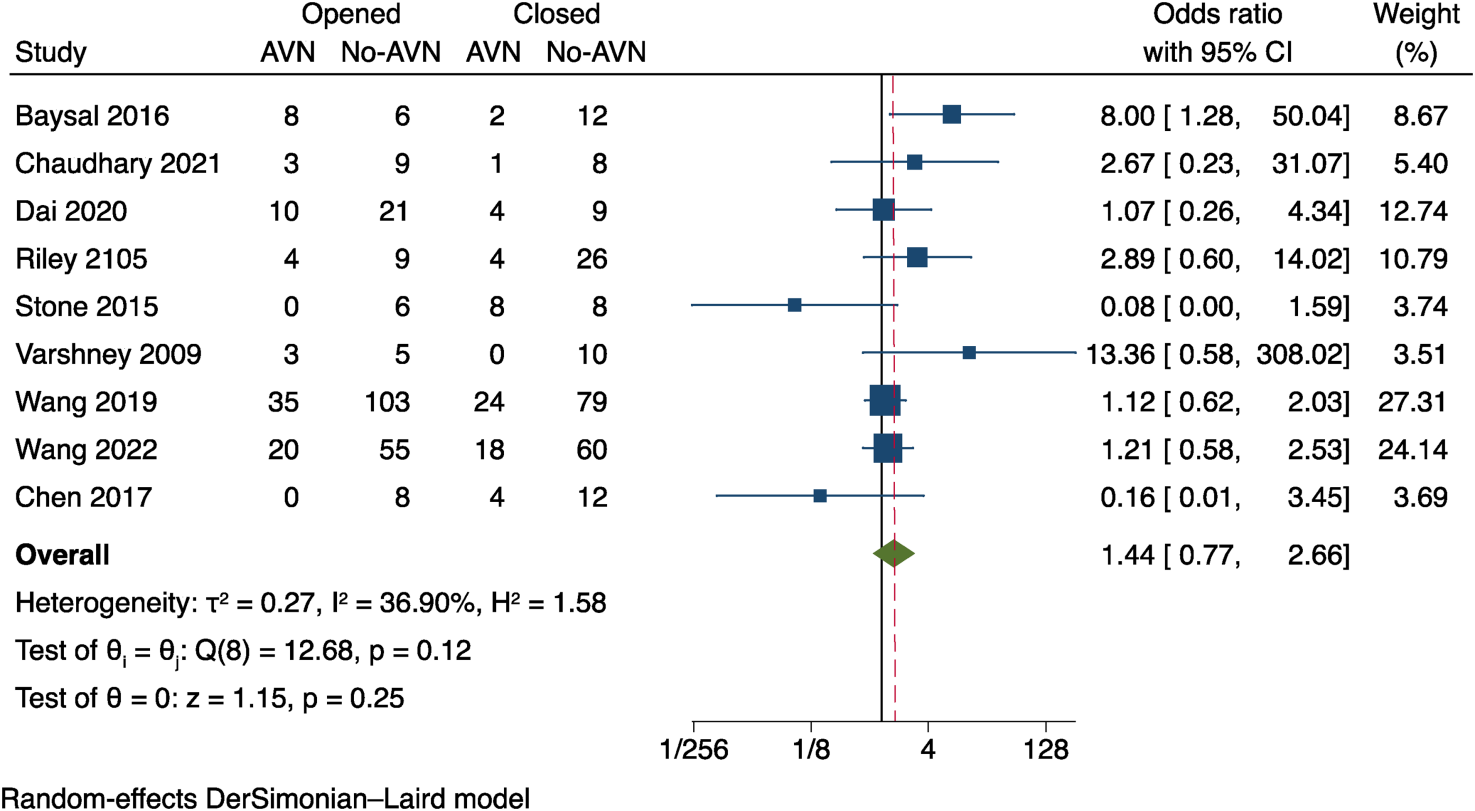
Results of a meta-analysis on the relationship between reduction techniques and postoperative AVN.

**“**Reduction methods” in medical literature refers to the two procedures of closed or opened reduction of fractures; data on the reduction methods from nine studies (10–13, 16–18, 20, 22) were available for Meta-analysis. Considering that the studies had a small amount of heterogeneity (*I*^2^ = 36.90%, *P* = 0.12), see Figure 6. The results showed: OR = 1.44, 95% CI: 0.77–2.66, *P* = 0.25. The two data groups did not differ statistically significantly from one another. This suggests no discernible difference in the effects of various fracture methods on postoperative AVN.

### Timing of the procedure

**Figure 7:**
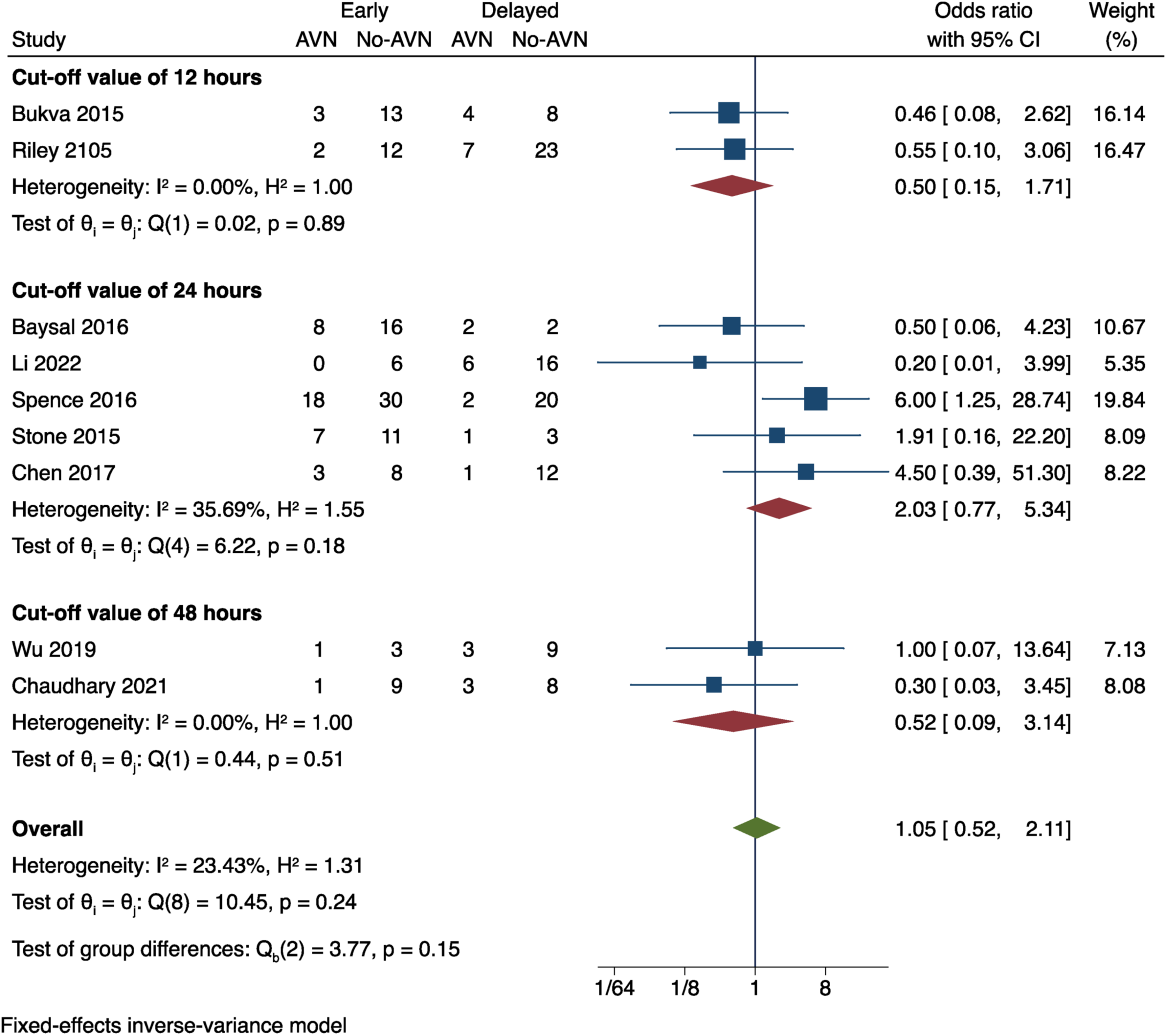
The correlation between the timing of the procedure and postoperative AVN was examined in a meta-analysis.

In nine studies, we investigated how the timing of the procedure of pediatric FNF impacted the risk of postoperative AVN. Differences in cut-off values for the timing of surgery, two studies (9, 20) used a cutoff of 48 h after the injury, respectively, In the remaining seven studies (13–16, 18, 21, 22), The cutoffs for the procedure timing were 12 and 24 h after the injury respectively, and subgroup analyses were done using such data. The outcomes of the three subgroups in the various effect models did not significantly differ. No significant statistical difference between the three groups was found in the results (OR = 1.05, 95% CI: 0.52–2.11, *P* = 0.15). These results imply that in children with internal fixated FNF, the procedure time does not significantly alter the risk of AVN (Figure 7).

### Injury mechanisms

**Figure 8:**
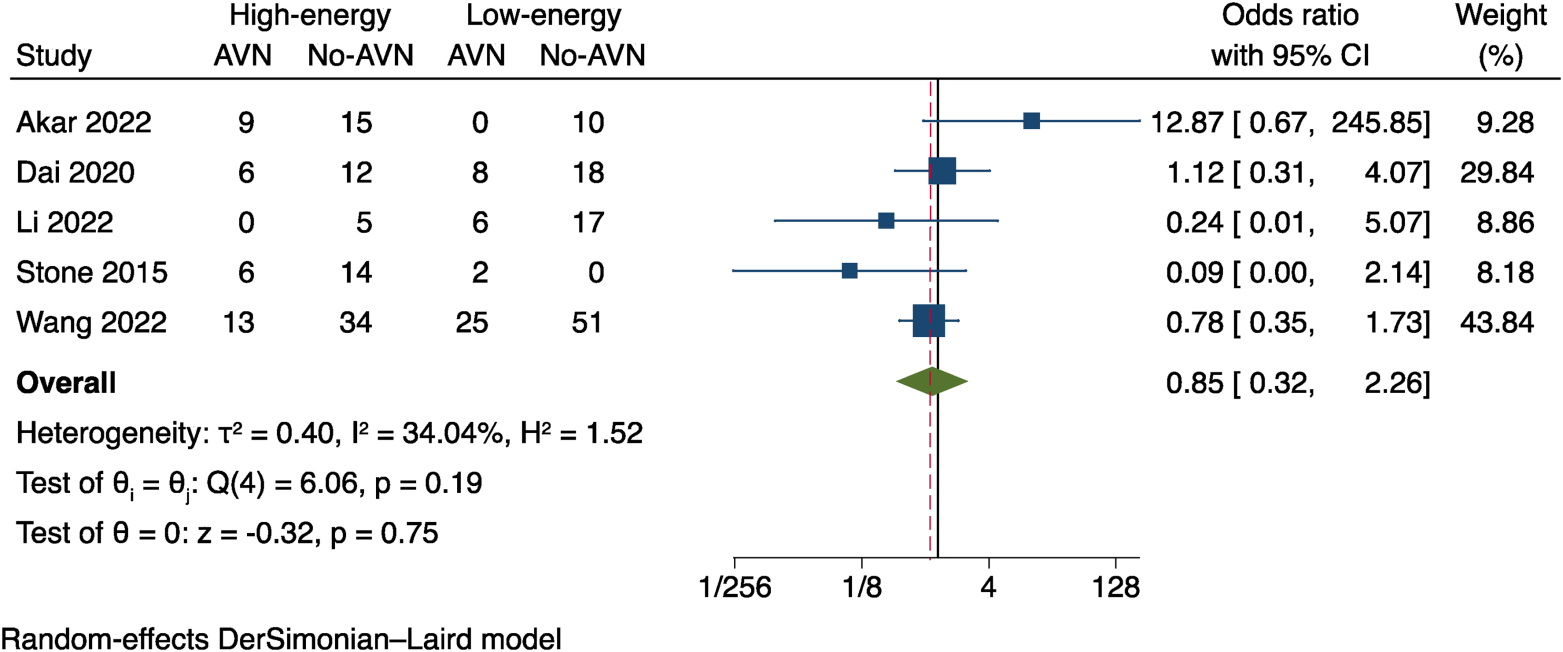
Results of a meta-analysis on the relationship between the mechanism of injury and postoperative AVN.

Data on the mechanism of injury was provided in 5 studies (11, 13, 17, 21, 23), and the data were sorted and transformed into dichotomous variables of high-energy injury (such as vehicle accidents and height-related incidents) and low-energy injury (such injuries during regular activities and simple falls) using an odds ratio (OR) for Meta-analysis. As that revealed mild heterogeneity between studies, the random effects model was preferred (*I*^2 ^= 34.04%, *P* = 0.19); see Figure 8. The outcomes were OR = 0.85, 95% CI: 0.32–2.26 and *P* = 0.75. Therefore, the impact of various fracture injury mechanisms on the incidence of postoperative AVN was not statistically different.

### The overall incidence of AVN

**Figure 9:**
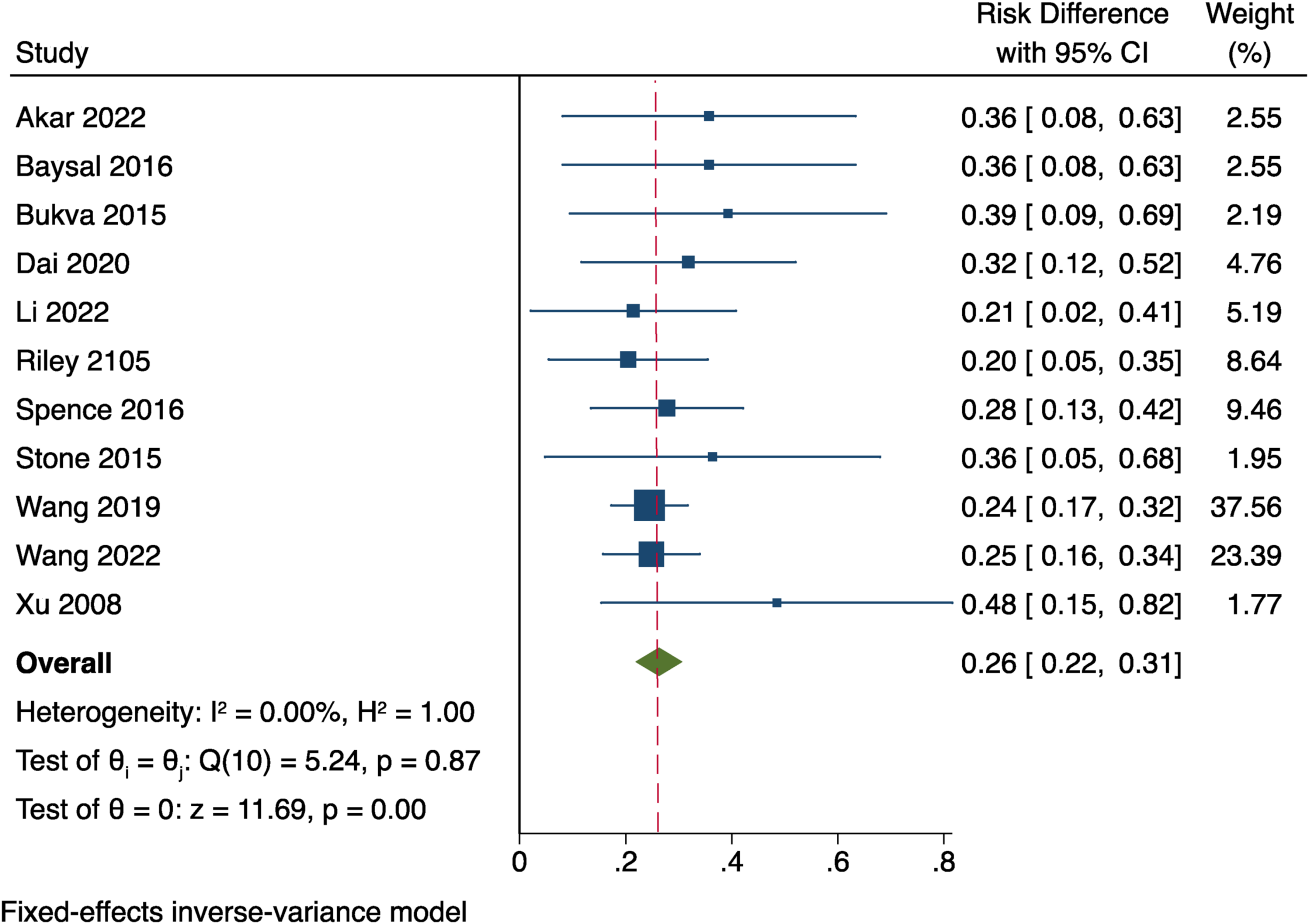
Results of a meta-analysis of postoperative AVN incidence overall.

Since the postoperative AVN meta-analysis of the 15 papers (11) with more than 5 cases demonstrated no heterogeneity between studies, the fixed effects model has been adopted (*P* = 0.87, *I*^2 ^= 0%). The incidence of AVN overall was 26% [95% CI (0.22–0.31)]. Look at Figure 9.

## Sensitivity analysis

**Figure 10:**
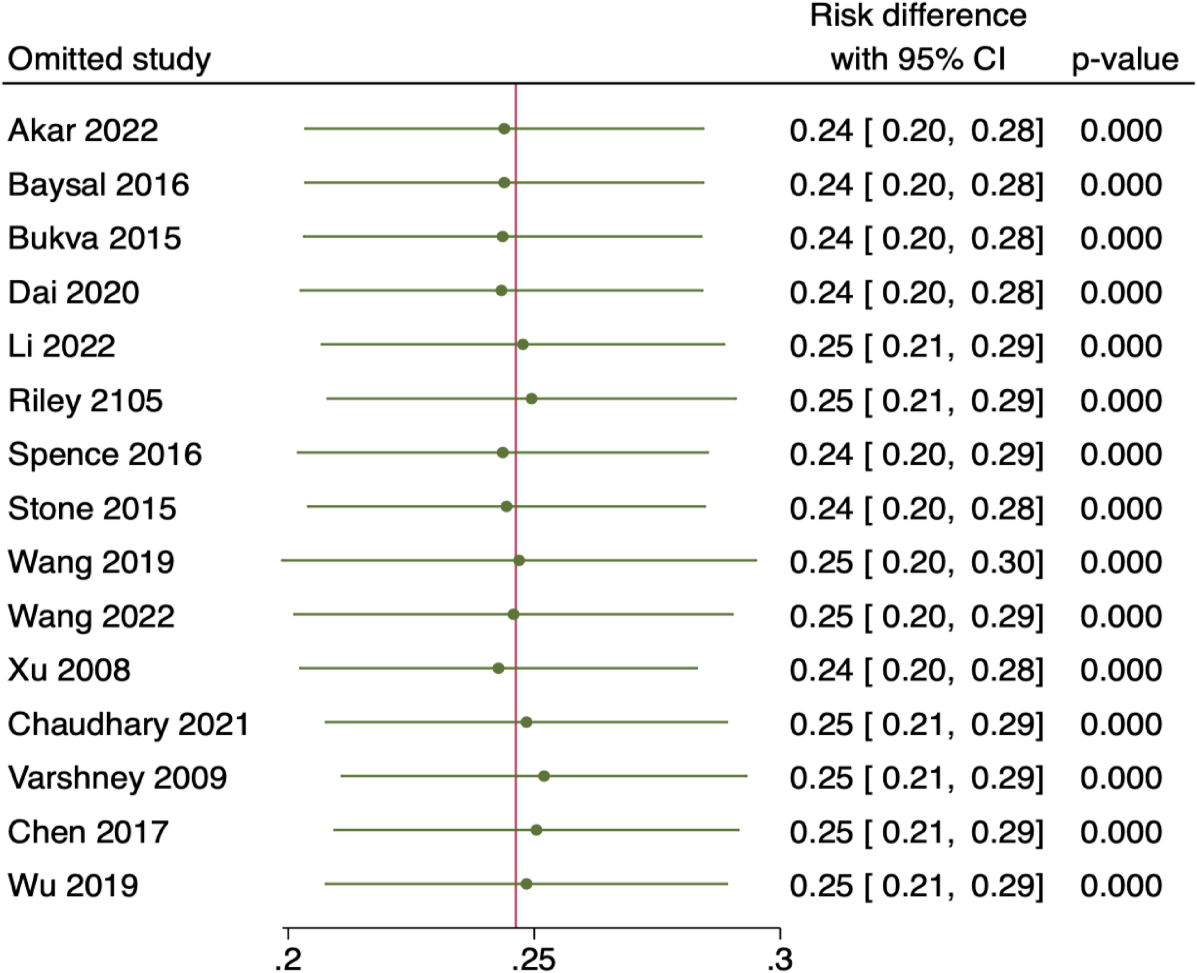
Sensitivity analysis utilising the leave-one-out approach.

The 15 included studies were eliminated for sensitivity analysis using the leave-one-out method. However, no specific studies with comparable findings were found that significantly altered the total effect size, demonstrating that the overall results were reliable and consistent. Look at Figure 10.

## Analysis of publication bias

**Figure 11:**
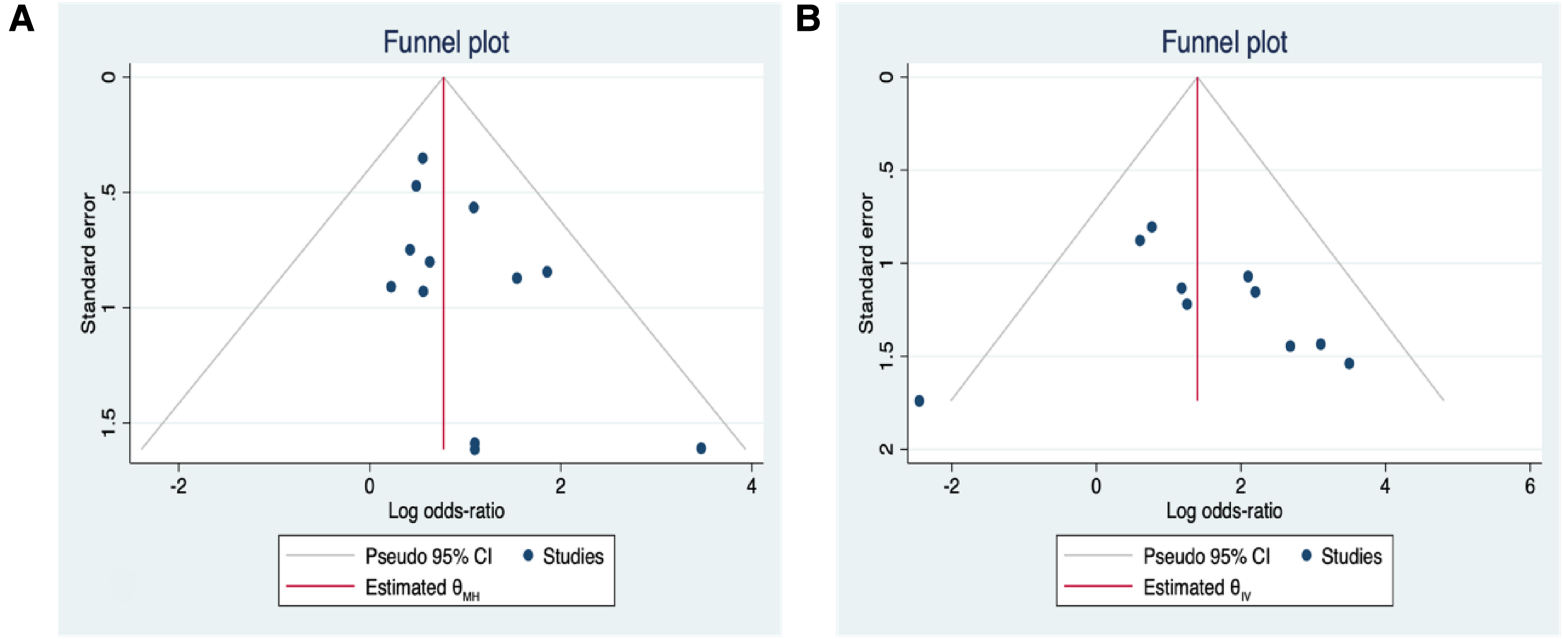
(**A**) Correlation funnel plot between postoperative AVN and delbet type of fractures; (**B**) initial fracture displacement and postoperative AVN in a funnel plot.

The results of the fracture Delbet type and initial fracture displacement funnel plots were utilised as an example to assess for publication bias in a meta-analysis of more than ten included studies (Figure 11). The included studies were mostly symmetrically distributed and fell within the 95% confidence interval, according to the funnel plots. Initial fracture displacement: Egger's test result: *t* = 0.50, *P* = 0.63, Begg's test result: *z* = 1.43, *P* = 0.15. Fracture Delbet type: *t* = 1.39, *P* = 0.19, Begg's test result: *z* = 1.71, *P* = 0.87.The findings revealed no consistent evidence of publication bias. Despite this, publication bias could not be assessed for each risk factor due to the scant amount of literature (less than 10) used to study the other risk variables. This calls for caution when using the study's findings in quotes.

## Discussion

In this study, we examined the risk variables for avascular necrosis (AVN) after internal fixation of children patients' FNF by performing a meta-analysis of 15 studies. To get accurate results, we carefully reviewed, examined, and incorporated past studies on AVN following the internal fixation of pediatric FNF. We included only high-quality research to address clinical questions about this illness. Our research indicates that age, Delbet type I/II fracture, initial fracture displacement, and relatively poor fracture reduction are important risk factors for postoperative AVN after internal fixation of pediatric FNF. Contrarily, the occurrence of AVN was unaffected by gender, the timing of fracture reduction, the mechanism of injury, or the method of fracture reduction. Furthermore, the funnel plot's visual data representation discovered no discernible publication bias.

Pediatric orthopaedic surgeons face enormous difficulty when treating FNF in children because of the severe dangers associated with post-operative AVN and non-union, which can endanger the child's health. However, more research is needed to understand how to treat these fractures fully. Incisional reduction is a common alternative to a closed reduction in cases where it fails. In particular, two or three hollow screws are suggested for internal fixation of Delbet I, II, and III fractures. To reduce vascular injury at the fracture site when closed reduction is impossible, conversion to opened reduction should be done immediately (24). Opened PHP plate internal fixation is often recommended for Delbet IV. It is essential to perform secure fixation of the fracture ends and anatomical reduction, whether closed or incisional internal fixation is utilised, to rebuild or protect the femoral head's fragile blood supply and prevent problems, including non-union and ischemia necrosis, As Tan et al. (25) concluded, the incidence of postoperative AVN is associated with anatomical reduction and secure fixation of the fracture end. Opened internal fixation is recommended to achieve this goal-the outcomes of studies by Bernstein (26), Barreto et al. (1), Tian et al. (27), Ju et al. (28), and Zheng et al. (29) are generally consistent with these findings (95% CI: 0.77–2.66, *P* = 0.25). The study's outcomes may have been biased by the included literature's inconsistent use of the incisional reduction approach and the operational variations amongst operators. In an attempt to explain the relationship between the selection of various incisional approaches and the occurrence of postoperative AVN, more research is necessary.

There is currently no consensus on whether the length of time between the onset of the fracture and surgery increases the incidence of postoperative AVN. Different studies use different time cutoffs, such as 12 h (15, 18), 24 h (13, 14, 16, 21, 22) and 48 h (9, 20), which generally suggests that the occurrence of AVN is positively correlated with the timing of the procedure, and early surgical treatment is recommended. However, a meta-analysis (30) showed that the occurrence of postoperative AVN is not related to the timing of surgery (≤24 h, >24 h).which is similar to the results of this meta-analysis. Given the numerous complicating factors, more research using large sample sizes is required to elucidate the correlation between postoperative AVN and the timing of the procedure.

For pediatric FNF, the Delbet categorisation system is extensively used, depending on the precise location of the fracture line, which divides the fractures into four categories: Type I is an epiphyseal separation through the growth plate, either with or without femoral head displacement from the acetabulum; Type II is transcervical; Type III is basicervical, and Type IV is intertrochanteric. Considering the anatomical characteristics of the blood supply to the femoral head (31–34), the growth plate's nutritional artery progressively communicates to the femoral head's ligament artery; as the kid grows and the growth plate ossifies, the growth plate becomes the main blood supply to the femoral head. The extracapsular arteries, on the other hand, play a gradually weakening or disappearing role until the growth plate is closed. The medial and lateral circumflex femoral arteries unite with the artery of the femoral head ligament to form a vascular network that delivers blood to the femoral head after passing through the ossified growth plate. Therefore, the insufficient blood supply to the femoral head, the joint capsule, and concomitant vascular obstruction may be the pathophysiological basis for avascular necrosis (AVN) post-fracture (35). Delbet Type I/II fractures meet the above two conditions. The analysis results of this study show that the overall trend in the incidence of postoperative AVN for the different Delbet types of fracture is that the incidence of postoperative AVN is highest for type II, lowest for type IV, and close for types I and III, but it is not clear which type of fracture is the independent risk factor.

The benefits of this meta-analysis study are as follows. First, Given the controversy surrounding the factors that may increase the risk of avascular necrosis (AVN), which will occur after internal fixation of pediatric FNF, this meta-analysis rigorously assessed any possible correlations among AVN and eight other indicators (such as gender, age, Delbet fracture type, etc.). Secondly, this meta-analysis's overall sensitivity analysis results are excellent, reasonable, and reliable.

## Limitations

Although this meta-overall analysis's sensitivity analysis was acceptable, as previously said, bias could not be completely ruled out. The following limitations may exist: (1) The research articles chosen for this analysis were released between 2008 and 2022. Most of these studies failed to account for confounding variables and thus could not report consistent impact factors. (2) Because selectable studies are all retrospective, the potential for bias remains. There were no randomised controlled studies, despite the thorough examination of the relevant literature in our analysis. (3) The original literature lacked several crucial evaluation indications, namely the number of internal fixations with hollow screws and access for incisional reduction. There was bias, and the related subgroup analysis could not be done. (4) The study only included references in Chinese and English. It was limited to published literature, subject to selection bias. In conclusion, the above limitations must be considered before adopting the results of this analysis.

## Conclusion

To sum up, this meta-analysis revealed that internal fixation for pediatric FNF results in a 26% overall incidence of avascular necrosis (AVN). Age, initial displacement, and poor reduction are risk factors for AVN. However, due to the limitations of this study and the use of univariate analysis, it is still being determined whether these three factors are independent risk factors for AVN undergoing internal fixation for pediatric FNF. In this study, gender, timing of the procedure, reduction methods, and injury mechanism were excluded from consideration as factors related to AVN after internal fixation for pediatric FNF. The overall trend in the incidence of postoperative AVN for the different Delbet types of fracture is that the incidence of postoperative AVN is highest for type II, lowest for type IV, and close for types I and III, but it is not clear which type of fracture is the independent risk factor.

## Data Availability

Publicly available datasets were analyzed in this study. This data can be found here: Wang WT, Li YQ, Guo YM, et al. Risk factors for the development of avascular necrosis after femoral neck fractures in children: a review of 239 cases. Bone Jt J 2019; 101-B(9): 1160–1167; doi: 10.1302/0301-620X.101B9.BJJ-2019-0275.R1.
